# Pregnancy Zone Protein as an Emerging Biomarker for Cardiovascular Risk in Pediatric Chronic Kidney Disease

**DOI:** 10.3390/jcm12185894

**Published:** 2023-09-11

**Authors:** Wei-Ling Chen, Wei-Ting Liao, Chien-Ning Hsu, You-Lin Tain

**Affiliations:** 1 Division of Pediatric Nephrology, Department of Pediatrics, Kaohsiung Chang Gung Memorial Hospital, Kaohsiung 833, Taiwan; weilingchen@cgmh.org.tw (W.-L.C.); weiting0307@cgmh.org.tw (W.-T.L.); 2 Department of Pharmacy, Kaohsiung Chang Gung Memorial Hospital, Kaohsiung 833, Taiwan; 3 School of Pharmacy, Kaohsiung Medical University, Kaohsiung 807, Taiwan; 4 College of Medicine, Chang Gung University, Taoyuan 330, Taiwan; 5 Institute for Translational Research in Biomedicine, Kaohsiung Chang Gung Memorial Hospital, Kaohsiung 833, Taiwan

**Keywords:** pregnancy zone protein, cardiovascular disease, chronic kidney disease, ambulatory blood pressure monitoring, children, congenital anomalies in the kidney and urinary tract (CAKUT), hypertension

## Abstract

Cardiovascular disease (CVD) is a significant cause of mortality and morbidity among children with chronic kidney disease (CKD). The causes of pediatric CKD differ from those in adults, as congenital anomalies in the kidney and urinary tract (CAKUT) are the leading causes in childhood. Identifying ideal markers of CVD risk early is crucial for CKD children to improve their care. Previously, we screened differentially expressed proteins in CKD children with or without blood pressure (BP) abnormalities and identified pregnancy zone protein (PZP). In 106 children and adolescents with CKD stages G1–G4, we analyzed plasma PZP concentration. The associations between PZP and ambulatory BP monitoring (ABPM) profile, parameters of cardiac and carotid ultrasounds, indices of arterial stiffness, and nitric oxide (NO) parameters were determined. We observed that PZP positively correlated with arterial stiffness indices, beta index, and pulse wave velocity in CAKUT. CKD children with abnormalities in ABPM and night dipping displayed a higher PZP concentration than those without. Additionally, the PZP level was positively correlated with NO bioavailability. In conclusion, our results suggest PZP has differential influences on cardiovascular risk in CAKUT and non-CAKUT children. Identification of this relationship is novel in the pediatric CKD literature.

## 1. Introduction

Despite substantial advances in the care of pediatric chronic kidney disease (CKD), the mortality rates remain high for children with CKD [1]. Cardiovascular diseases (CVD) are the most frequent cause of morbidity and mortality in childhood CKD [2]. Similar to adults, children with CKD have a high prevalence of traditional and uremia-related CVD risk factors [2,3]. Unlike in adults, congenital anomalies in the kidney and urinary tract (CAKUT) are the most common etiologies in pediatric CKD [4]. Several risk factors for CAKUT are interconnected with adulthood hypertension and CVD [4,5]. Recent evidence supports the notion that identifying childhood CVD risk factors early may aid pediatricians in making early diagnoses, initiating preventive interventions, and monitoring for complications in pediatric CKD.

Hypertension is a well-established risk factor linked to CVD as well as CKD [6]. Ambulatory blood pressure monitoring (ABPM) is the gold standard for diagnosing pediatric hypertension. We and others have shown that up to 50% of CKD children displayed abnormalities in ABPM [7,8]. Additionally, several noninvasive structural and functional assessments have been utilized to better define the presence of early CVD, covering left ventricular (LV) mass, LV mass index (LVMI), carotid intimal and medial thickness (cIMT), augmentation index, beta index, and pulse wave velocity (PWV), [9,10]. As CAKUT develops early in life and may progress to end-stage kidney disease (ESKD) in early adulthood, early detection of the above-mentioned surrogate markers may help reduce cardiovascular mortality and morbidity in CKD.

Also, biomarkers are perceived as surrogates for CV outcomes [5,11]. Although many biomarkers have been identified in CKD, merely a few of them have been examined in childhood CKD, particularly focusing on CVD risk [5,12,13]. 

A broad panel of biomarkers has shed light on the diagnosis, risk, and prognosis of cardiorenal syndrome characterized by the complex interaction between kidney and cardiovascular pathogenesis [14]. Accordingly, the identification of accurate biomarkers can be helpful in various aspects of CKD treatment, including the prevention of CVD events. Our prior work indicated that nitric oxide (NO)-related parameters participate in BP abnormalities in ABPM in CKD children [8]. Currently, proteomics research has been broadly employed to identify protein biomarkers in many diseases [15]. Our previous study using a proteomic approach identified 20 differentially expressed proteins between CKD children with CAKUT or non-CAKUT and with or without ABPM abnormalities [16]. Our findings identified pregnancy zone protein (PZP), which will be validated in the current study to elucidate its association with CV risk in CKD children.

PZP, a gene located on chromosome 12p13.31, is an α2 glycoprotein with a molecular weight of 360 kDa [17]. The amino acid composition of PZP is similar to that of α2-macroglobulin [18]. PZP is a broad-spectrum immunosuppressant [19] with antiproteinase activity. During pregnancy, the plasma levels of PZP increase rapidly in the first trimester and may reach concentrations of around 1000 μg/L in the third trimester [20]. The upregulation of PZP represents a major maternal adaptation and the low placental expression of PZP is related to preeclampsia [21]. Currently, there is scanty information on the utility of PZP as a biomarker in the pediatric population [22]. Although prior work revealed that PZP is a potential biomarker for early-onset myocardial infarction [23], it has never previously been studied in the context of CVD in CKD. Our primary objective was to assess the association between PZP and ABPM abnormalities, parameters of cardiac and carotid ultrasounds, and indices of arterial stiffness in children with CKD. The secondary objective was to find a relation between the PZP and NO pathway in these patients.

## 2. Materials and Methods

### 2.1. The Study Group

In this cross-sectional study, we analyzed data from 106 children and adolescents with CKD who received the measurement of PZP. Our data were obtained from a prospective cohort study (Precision Medicine Project) approved by the ethics committee of Chang Gung Medical Foundation, Taoyuan, Taiwan (201701735A3C501 and 202001973A3C601). The enrollment took place between November 2018 and April 2022. Patients between 6 and 18 years of age with CKD stages 1–4 attending the Pediatric Nephrology Clinic at Kaohsiung Chang Gung Memorial Hospital were enrolled. All participants and parents gave their informed consent in writing before being included in the investigation. CKD is defined according to KDIGO [24]. The estimated glomerular filtration rate (eGFR) was calculated based on the Schwartz formula [25]. CKD was classified according to the GFR G1–G5 category as G1 ≥ 90, G2 60–89, G3 30–59, G4 15–29, and G5 < 15 mL/min/1.73 m^2^. The etiologies of CKD were classified based on the presence or absence of CAKUT. CAKUT covered renal agenesis, kidney hypo-/dysplasia, multi-cystic kidney dysplasia, duplex collecting system, posterior urethral valves, horseshoe kidney, and ureter abnormalities [4]. The exclusion criteria consist of CKD stage G5, pregnancy, congenital heart disease, dialysis or kidney transplant, and uncooperative patients. 

Venous peripheral blood was collected after overnight fasting and refrigerated immediately before being stored at −80 °C freezer. Blood urea nitrogen, creatinine, total cholesterol, low-density lipoprotein, triglyceride, hemoglobin, hematocrit, sodium, potassium, glucose, calcium, phosphate, and uric acid were analyzed by the hospital central laboratory as described before [8]. The total protein-to-creatinine ratio was evaluated in on-the-spot urine samples.

### 2.2. BP Measurement and Cardiovascular Assessment

BP was measured using an oscillometric device at the clinic visit. Hypertension is defined according to the 2017 American Academy of Pediatrics (AAP) guidelines [26]. A subgroup of 62 participants who were aged 6 years or over received CV assessment and ABPM analysis. 

ABPM with Oscar II monitoring device (SunTech Medical, Morrisville, NC, USA) and cuff of appropriate size were performed as described previously [8]. Abnormalities in ABPM included (1) awake, asleep, systolic, or diastolic BP loads ≥ 95th percentile for height and sex; (2) awake, asleep, systolic, or diastolic BP load ≥ 25%; and (3) non-dipping systolic and diastolic BP patterns were defined as a percentage dipping < 10% [27].

A cardiac ultrasound study was carried out using a Philips IE33 ultrasound (Bothell, WA, USA) to assess LV mass. The LVMI was calculated by indexing LV mass to height^2.7^ [28]. Carotid ultrasound was performed by an ALOKA ProSound α7 ultrasound (Aloka Co., Tokyo, Japan) to detect cIMT. PWV, augmentation index, and beta index were analyzed by means of echo-tracking methods (e-TRACKING system; Aloka Co., Tokyo, Japan).

### 2.3. Measurement of PZP and NO Parameters 

The PZP concentrations in plasma were determined in duplicates using an enzyme-linked immunosorbent assay (CSB-EL019131HU, Cusabio, Houston, TX, USA). The optical density was determined at 450 nm. The coefficient of variation was <15%. Assays were performed according to the manufacturer’s instructions.

An ancillary study was conducted on a subgroup of 42 subjects who had concurrent laboratory tests of PZP and NO-related parameters. L-arginine is the substrate of NO synthase (NOS), while methylated arginine derivatives, asymmetric and symmetric dimethylarginine (ADMA, SDMA), are endogenous NOS inhibitors. Plasma L-arginine, ADMA, and SDMA concentrations were quantified using high-performance liquid chromatography (HPLC) using homoarginine as an internal standard [8]. These NO-related parameters were derivatized with o-phthaldialdehyde containing 3-mercaptopropionic, separated by means of reverse-phase chromatography, and analyzed with fluorescence detection. The ratio of L-arginine to ADMA was calculated as it has been considered an index of NO bioavailability [29].

### 2.4. Statistical Analysis

Statistical elaboration was performed using Statistical Package for the Social Sciences (SPSS) software 14.0 (Chicago, IL, USA). Descriptive statistics are reported as median and interquartile ranges (or ranges) for continuous variables, while categorical variables are described as numbers and percentages. The following tests were used (depending on data distribution): Mann–Whitney *U* test, Spearman rank correlation, Chi-square test, and Fisher’s exact test. Multiple regression analysis revealed independent risk factors. A *p*-value < 0.05 was considered statistically significant.

## 3. Results

### 3.1. Cohort Characteristics

The characteristics of the study participants are illuminated in Table 1. In total, there were 106 children and adolescents with CKD stages G1–G4. Our study population had a median age of 9.8 years, 56.2% male, and 67.9% with CAKUT. The majority (76/106, 71.7%) of our study population was in CKD stage G1 and the minority was in G4 (2/106, 1.9%). Most subjects recruited in the current study fall into the early stages of CKD. The diagnosis of hypertension accounting for 42.5% of CKD children and adolescents was made by office BP readings. Compared with CAKUT, children in the non-CAKUT group displayed heavier proteinuria and had higher plasma concentrations of total cholesterol and low-density lipoprotein, but lower calcium concentrations.

### 3.2. Plasma Pregnancy Zone Protein Concentration

**Figure 1:**
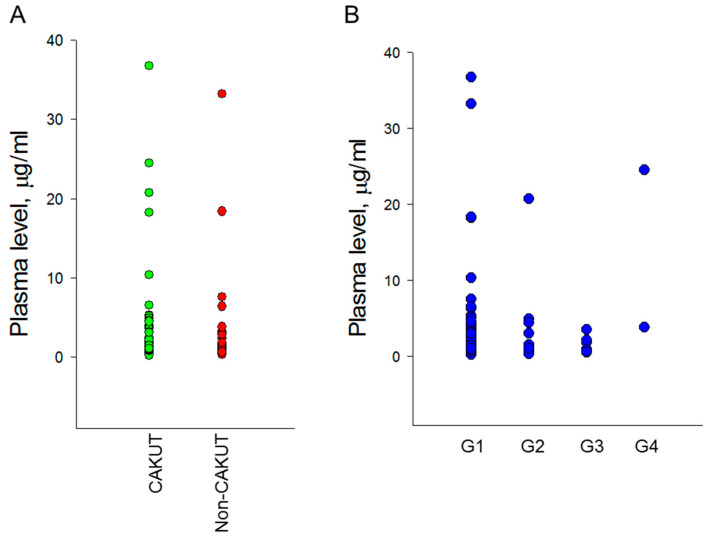
Comparison of plasma pregnancy zone protein concentrations between (**A**) the CAKUT and non-CAKUT group and between (**B**) the CKD stages G1-G4 group.

We analyzed the plasma concentrations of pregnancy zone protein in children with CKD. Circulating PZP concentration was compared with etiologies of CKD and different stages of CKD. Figure 1A illustrates that there is no difference in the plasma concentration of PZP between the CAKUT and non-CAKUT groups (*p* = 0.288). Additionally, differences in the PZP concentration according to the G1-G4 stages of CKD did not exist (Figure 1B, *p* = 0.167). We did not find any significance between PZP with plasma Cr concentration (*p* = 0.136) and eGFR (*p* = 0.808). An association was revealed between PZP with systolic BP (*r* = 0.198, *p* = 0.047). The correlation between PZP and systolic BP becomes more significant in the CAKUT group (*r* = 0.344, *p* = 0.004), while it shows no significance in the non-CAKUT group (*r* = -0.124, *p* = 0.485). 

### 3.3. Cardiovascular Assessment

In a subgroup of 62 pediatric patients with CKD who had concurrently received a comprehensive CV assessment, we found that the LV mass and LVMI did not differ between the two different groups in the presence or absence of CAKUT. Likewise, the cIMT in the CAKUT group is comparable to that in the non-CAKUT group. We also utilized several indices to evaluate arterial stiffness, including the beta index, augmentation index, and PWV. No differences in these indices between the two groups were revealed (Table 2).

Table 3 illustrates correlations between plasma PZP concentrations with CV markers. Spearman’s rank correlation analysis shows that PZP is positively correlated with left ventricular mass (*r* = 0.335, *p* = 0.008), irrespective of the CKD etiologies. In the CAKUT group, PZP is correlated with beta index (*r* = 0.326, *p* = 0.043) and PWV (*r* = 0.384, *p* = 0.016). PZP is correlated with left ventricular mass (*r* = 0.476, *p* = 0.022) in children with non-CAKUT. In multiple regression analysis for PWV, PZP was demonstrated to be an independent risk factor (*p* = 0.042), controlling for age, sex, and eGFR.

### 3.4. Plasma PZP Concentration vs. ABPM Profile

Table 4 demonstrates that a total of 35 pediatric patients with CKD received an ABPM study. We observed that 71.4% of them (25/35) had at least one ABPM abnormality, including 6 participants (17.1%) with 24 h BP ≥ 95th percentile, 4 participants (11.4%) with daytime BP ≥ 95th percentile, 9 participants (25.7%) with nighttime BP ≥ 95th percentile, 17 participants (48.6%) with BP load ≥ 25th percentile, and 19 participants (54.3%) with a nocturnal decrease in BP load < 10%.

In the CAKUT group, plasma PZP concentration was significantly higher in children with CKD accompanied by a nocturnal non-dipping status and ABPM profile above the goal. Analysis of children with non-CAKUT CKD did not show any significant difference in plasma PZP concentration between normal and abnormal ABPM profiles (Table 4). Additionally, PZP was associated with an abnormal ABPM profile (*p* = 0.034) in the adjusted model controlling for age, sex, and eGFR.

Next, we analyzed the associations between PZP concentrations and NO-related parameters. The plasma PZP level was negatively associated with ADMA (*r* = −0.474, *p* = 0.035), but positively associated with AAR (*r* = 0.445, *p* = 0.049). No associations exist between plasma PZP concentration and L-arginine as well as SDMA. Furthermore, the patients were divided into two groups based on the PZP concentration (Table 5). Patients with high PZP levels had significantly lower ADMA (*p* = 0.026) and higher AAR (*p* = 0.036). Our data suggest a link between PZP and NO bioavailability.

## 4. Discussion

This is, to our knowledge, the first study comparing PZP as a protein biomarker with CVD structural and functional markers, ABPM profile, and NO parameters in pediatric CKD. Our major findings are as follows: (1) PZP concentration was not influenced by the etiologies or severity of CKD; (2) up to 70% of CKD children had an abnormal ABPM profile; (3) PZP was positively correlated with beta index and PWV in CAKUT; (4) CKD children with a night dipping and ABPM profile above the goal had a higher PZP concentration than those without; and (5) CKD children with high PZP levels displayed low ADMA and high AAR.

This is in line with prior work revealing that certain CVD risk markers already appear in children with early stages of CKD, for example, abnormalities in ABPM [6,8,10,11,16]. Although the severity of CKD was reported to be associated with high PWV and LV mass [8], we observed that these CV markers were not different between the two groups classified by the presence or absence of CAKUT. Compared with 42.5% of CKD children diagnosed with hypertension by means of office BP, more than 70% of them had BP abnormalities in ABPM. The high prevalence of abnormalities in ABPM agrees with the idea that ABPM may detect hypertension and predict CV risk better than office BP, especially in CKD children [30].

Using a proteomic approach, we screened potential protein biomarkers between the groups of CKD children for the presence or absence of abnormalities in ABPM [16]. In agreement with our former research, we identified PZP-linking CVD risk markers and found that they might have a differential impact on the two different CKD groups with or without CAKUT. Our results revealed that PZP concentration was positively correlated with systolic BP, beta index, PWV, and BP abnormalities in ABPM only becoming significant in the CAKUT group, instead of the non-CAKUT group. It is highly uncertain whether elevations in PZP are helpful or detrimental to CV health. So far, most PZP studies have been focused on pregnant women [18,21,31]. It has been proposed that low PZP levels during gestation may raise the risk of placental dysfunction [32]. In the pediatric population, only one study reported that PZP levels were elevated in growth hormone deficiency patients [22].

Previous research suggests that CAKUT is associated with longer survival owing to lower CV mortality [32]. Hence, elevations in PZP might be a compensatory mechanism to avert CKD children with CAKUT from subclinical CVD and BP abnormalities. NO is a well-known vasodilator. High AAR and low ADMA represent increased NO bioactivity in favor of vasodilation. Considering that high PZP correlates to high AAR and low ADMA, PZP may mediate the NO pathway to induce a compensatory response in pediatric patients with early stages of CKD following the rise in BP. Animal data showing that elevations in PZP levels in developing kidneys coincide with those with hypertension also add credence to some of these observations [33]. In a maternal NO deficiency rat model, the renal abundance of PZP increased 11.13 fold in neonatal rats. As adult offspring developed hypertension and renal hypertrophy in response to deficient NO in early life, the increases in PZP might be a link between NO deficiency and hypertension later in life. On the other hand, PZP concentration was only positively associated with LV mass, but not other markers in the non-CAKUT group. Together, whether PZP may have differential impacts on the development of CVD in CKD children with vs. without CAKUT awaits further elucidation.

Proteomics-based approaches have given valuable information about protein contents and discovered several disease-specific biomarkers in CKD [34]. However, most studies have been conducted on adults, and proteomics research in CKD has not been extensively transferred to the pediatric population [5]. This is the first study using PZP from proteomic profiling data to describe the upregulation of PZP in association with CVD markers, which supports the possibility that protein biomarkers may have clinical usefulness for discriminating CV risk in pediatric CKD [35,36].

We acknowledge that our study has the following limitations: First, the number of participants recruited in our study is small, which might not provide sufficient power to determine slight differences and represent an entire population. More studies with larger cohorts are required to confirm our observations. Second, we examined the associations between PZP and CVD markers at different stages of CKD without recruiting normal or hypertensive controls. Although the plasma level of PZP in the current study is comparable to that earlier reported in children [22], whether the level differs between children with and without CKD or between hypertensive children with and without CKD remains to be elucidated. Last, this is a cross-sectional study. It is not possible to determine the cause and effect, but it is possible to describe the associations of these findings in children with CKD. Additional studies are required to elucidate the role of PZP in CVD as well as the NO signaling pathway.

## 5. Conclusions

In conclusion, we described that in CKD children with CAKUT, PZP concentration positively correlated with systolic BP, beta index, PWV, and BP abnormalities in ABPM. Although the mechanism underlying how PZP affects CV outcomes in pediatric patients with CKD has not yet been elucidated, our results offer new insights into the links between PZP, CVD markers, and NO in children with early stages of CKD. Better insight into the impact of PZP on CV risk in childhood CKD would help us to initiate early treatment to help CKD children who are at risk of CVD.

## Figures and Tables

**Table 1:** Cohort characteristics of the study population (*n* = 88).

Characteristics	Overall	CAKUT	Non-CAKUT
	N = 106	N = 72	N = 34
Age, years	9.8 (6.3–13.1)	9.05 (5.72–13)	10.7 (7.4–13.8)
Male	59 (56.2%)	43 (59.7%)	16 (47.1%)
CKD stage			
Stage G1	76 (71.7%)	49 (68.1%)	27 (79.4%)
Stage G2	20 (18.9%)	17 (23.6%)	3 (8.8%)
Stage G3	8 (7.5%)	5 (6.9%)	3 (8.8%)
Stage G4	2 (1.9%)	1 (1.4%)	1 (2.9%)
Body height, percentile	50 (15–85)	50 (15–82.5)	50 (25–85)
Body weight, percentile	50 (25–85)	50 (15–85)	50 (25–88)
Body mass index (kg·m^−2^)	17.5 (15.3–20.5)	16.5 (14.9–20.1)	18.7 (15.7–21.7)
Systolic blood pressure (mmHg)	112 (102–123)	112 (102–125)	110 (101–120)
Diastolic blood pressure (mmHg)	72 (66–78)	71 (66–78)	72 (67–79)
Hypertension (by means of office BP)	45 (42.5%)	31 (43.1%)	14 (41.2%)
BUN (mg/dL)	13 (11–16)	13 (11–16)	12 (10.5–16.5)
Creatinine (mg/dL)	0.55 (0.45–0.68)	0.56 (0.46–0.7)	0.53 (0.43–0.67)
eGFR (mL/min/1.73 m^2^)	100.7 (88.2–114.6)	96.7 (84.3–107.1)	110.1 (90.4–124.8)
UTCR (mg/g)	67.8 (39.9–182.7)	61.6 (37–99.6)	141.2 (52.3–818.6) *
Hemoglobin (g/dL)	13.5 (13–14.1)	13.6 (13.1–14.3)	13.2 (12.4–13.9)
Hematocrit (%)	40 (38.6–41.8)	40.4 (38.5–42.3)	39.4 (37.3–41.2)
Fasting glucose (mg/dL)	88 (83–92)	88 (83–91)	87 (83–93)
Total cholesterol (mg/dL)	175 (149–207)	167 (146–194)	191 (151–216) *
Low-density lipoprotein (mg/dL)	95 (78–119)	90 (77–113)	109 (81–133) *
Triglyceride (mg/dL)	67 (45–94)	65 (45–92)	70 (46–112)
Uric acid (mg/dL)	5.15 (4.4–6.25)	5.1 (4.53–6.08)	5.2 (4.1–6.4)
Sodium (mEq/L)	140 (139–141)	140 (139–142)	140 (139–141)
Potassium (mEq/L)	4.4 (4.2–4.6)	4.4 (4.2–4.6)	4.4 (4.1–4.6)
Calcium (mg/dL)	9.9 (9.7–10.2)	10 (9.8–10.3)	9.7 (9.4–10) *
Phosphate (mg/dL)	4.9 (4.6–5.2)	4.9 (4.6–5.2)	4.8 (4.6–5.2)

Data presented as medians (IQR) or *n* (%). BUN = blood urea nitrogen; eGFR = estimated glomerular filtration rate; UTCR = urine total protein-to-creatinine ratio; CAKUT = Congenital anomalies in the kidneys and urinary tract. * *p* < 0.05 by the Mann–Whitney *U* test.

**Table 2:** Cardiovascular assessments of the study population.

CV Markers	CAKUT	Non-CAKUT
	*n* = 39	*n* = 23
Left ventricular mass (g)	80.1 (58.6–98.5)	85.9 (61.5–101)
LVMI (g/m^2.7^)	25.3 (22.5–31.9)	30.1 (23.1–38.8)
cIMT	0.38 (0.34–0.42)	0.34 (0.31–0.42)
Beta index	3.5 (2.6–4.3)	3.4 (3.1–3.8)
Augmentation index	−1.8 (−10.5–6)	0.9 (−5–5.9)
PWV	4 (3.4–4.6)	4 (3.7–4.2)

Data are medians (IQR). LVMI = left ventricular mass index. cIMT = carotid artery intima-media thickness. LVMI = left ventricular mass index. PWV = pulse wave velocity.

**Table 3:** Correlation between plasma pregnancy zone protein concentrations and cardiovascular markers.

Cardiovascular Markers	Overall (*n* = 62)		CAKUT (*n* = 39)		Non-CAKUT (*n* = 23)	
	*r*	*p*	*r*	*p*	*r*	*p*
Left ventricular mass	0.335	0.008 *	0.262	0.107	0.476	0.022 *
LVMI	0.063	0.631	0.048	0.774	0.108	0.625
cIMT	−0.145	0.259	−0.308	0.056	−0.033	0.88
Beta index	0.157	0.222	0.326	0.043 *	−0.211	0.333
Augmentation index	−0.143	0.267	−0.099	0.548	−0.071	0.749
PWV	0.218	0.089	0.384	0.016 *	−0.167	0.446

* *p* < 0.05 by Spearman’s correlation coefficient.

**Table 4:** Plasma PZP concentrations vs. ABPM profile.

ABPM Profile	*n*	CAKUT	*n*	Non-CAKUT
	21		14	
24 h BP				
Normal	18	1.83 (0.27–33.2)	11	0.78 (0.31–3.79)
Abnormal	3	2.15 (1.77–3.58)	3	0.87 (0.71–1.87)
Daytime BP				
Normal	20	2.09 (0.27–33.2)	11	0.78 (0.31–3.79)
Abnormal	1	1.77	3	0.87 (0.71–1.87)
Nighttime BP				
Normal	15	2.03 (0.27–18.2)	11	0.78 (0.31–3.79)
Abnormal	6	1.96 (1.14–33.2)	3	0.87 (0.71–1.87)
BP load				
Normal	12	1.64 (0.27–18.2)	6	0.92 (0.32–3.08)
Abnormal	9	2.15 (1.14–33.2)	8	0.79 (0.31–3.79)
Night dipping				
Normal	8	1.19 (0.38–3.58)	8	0.92 (0.32–3.08)
Abnormal	13	2.52 (0.27–33.2) *	6	0.79 (0.31–3.79)
Total ABPM profile				
Normal	5	0.86 (0.38–2.03)	5	1.06 (0.32–3.08)
Abnormal	16	2.42 (0.27–33.2) *	9	0.71 (0.31–3.79)

Data are medians (range). * *p* < 0.05 by the Mann–Whitney *U* test.

**Table 5:** Plasma PZP Concentrations vs. NO-related Parameters.

NO-Related Parameters	PZP (<Median)	PZP (>Median)
	*n* = 21	*n* = 21
L-arginine (μM)	116 (77–103.6)	122.7 (83.6–166.8)
ADMA (μM)	0.66 (0.35–1.49)	0.58 (0.34–1) *
SDMA (μM)	0.32 (0.24–0.45)	0.3 (0.23–0.66)
AAR (μM/μM)	168.9 (94–298.1)	204.5 (83.4–357.5) *

Data are medians (range). * *p* < 0.05 by the Mann–Whitney *U* test.

## Data Availability

The data that support the findings of this study are contained within the article.

## References

[1] Collins A.J., Foley R.N., Gilbertson D.T., Chen S.C. (2015). United States Renal Data System public health surveillance of chronic kidney disease and end-stage renal disease. Kidney Int. Suppl..

[2] Mitsnefes M.M. (2012). Cardiovascular disease in children with chronic kidney disease. J. Am. Soc. Nephrol..

[3] London G.M., Marchais S.J., Metivier F., Guerin A.P. (2000). Cardiovascular risk in end-stage renal disease: Vascular aspects. Nephrol. Dial. Transplant..

[4] Renkema K.Y., Winyard P.J., Skovorodkin I.N., Levtchenko E., Hindryckx A., Jeanpierre C., Weber S., Salomon R., Antignac C., Vainio S. (2011). Novel perspectives for investigating congenital anomalies of the kidney and urinary tract (CAKUT). Nephrol. Dial. Transplant..

[5] Tain Y.L., Hsu C.N. (2022). Cardiovascular Risks of Hypertension: Lessons from Children with Chronic Kidney Disease. Children.

[6] Vidi S.R. (2018). Role of hypertension in the progression of chronic kidney disease in children. Curr. Opin. Pediatr..

[7] Mitsnefes M., Flynn J., Cohn S., Samuels J., Blydt-Hansen T., Saland J., Kimball T., Furth S., Warady B., CKiD Study Group (2010). Masked hypertension associates with left ventricular hypertrophy in children with CKD. J. Am. Soc. Nephrol..

[8] Hsu C.N., Lu P.C., Lo M.H., Lin I.C., Tain Y.L. (2019). The association between nitric oxide pathway, blood pressure abnormalities, and cardiovascular risk profile in pediatric chronic kidney disease. Int. J. Mol. Sci..

[9] Urbina E.M., Williams R.V., Alpert B.S., Collins R.T., Daniels S.R., Hayman L., Jacobson M., Mahoney L., Mietus-Snyder M., Rocchini A. (2009). Noninvasive assessment of subclinical atherosclerosis in children and adolescents: Recommendations for standard assessment for clinical research: A scientific statement from the American Heart Association. Hypertension.

[10] Shroff R., Dégi A., Kerti A., Kis E., Cseprekál O., Tory K., Szabó A.J., Reusz G.S. (2013). Cardiovascular risk assessment in children with chronic kidney disease. Pediatr. Nephrol..

[11] De Ferranti S.D., Steinberger J., Ameduri R., Baker A., Gooding H., Kelly A.S., Mietus-Snyder M., Mitsnefes M.M., Peterson A.L., St-Pierre J. (2019). Cardiovascular risk reduction in high-risk pediatric patients: A scientific statement from the American Heart Association. Circulation.

[12] Greenberg J.H., Kakajiwala A., Parikh C.R., Furth S. (2018). Emerging biomarkers of chronic kidney disease in children. Pediatr. Nephrol..

[13] Sandokji I., Greenberg J.H. (2021). Plasma and Urine Biomarkers of CKD: A Review of Findings in the CKiD Study. Semin. Nephrol..

[14] Gembillo G., Visconti L., Giusti M.A., Siligato R., Gallo A., Santoro D., Mattina A. (2021). Cardiorenal Syndrome: New Pathways and Novel Biomarkers. Biomolecules.

[15] Siwy J., Mischak H., Zürbig P. (2019). Proteomics, and personalized medicine: A focus on kidney disease. Expert Rev. Proteom..

[16] Chen W.L., Tain Y.L., Chen H.E., Hsu C.N. (2021). Cardiovascular Disease Risk in Children With Chronic Kidney Disease: Impact of Apolipoprotein C-II and Apolipoprotein C-III. Front. Pediatr..

[17] Smithies O. (1959). Zone electrophoresis in starch gels and its application to studies of serum proteins. Adv. Protein Chem..

[18] Devriendt K., Van den Berghe H., Cassiman J.J., Marynen P. (1991). Primary structure of pregnancy zone protein. Molecular cloning of a full-length PZP cDNA clone by the polymerase chain reaction. Biochim. Biophys. Acta.

[19] Stimson W.H. (1980). Are pregnancy-associated serum proteins responsible for the inhibition of lymphocyte transformation by pregnancy serum?. Clin. Exp. Immunol..

[20] von Schoultz B. (1974). A quantitative study of the pregnancy zone protein in the sera of pregnant and puerperal women. Am. J. Obstet. Gynecol..

[21] Löb S., Vattai A., Kuhn C., Mittelberger J., Herbert S.L., Wöckel A., Schmoeckel E., Mahner S., Jeschke U. (2022). The Pregnancy Zone Protein (PZP) is significantly downregulated in the placenta of preeclampsia and HELLP syndrome patients. J. Reprod. Immunol..

[22] Devriendt K., Massa G., de Zegher F., Vanderschueren-Lodeweyckx M., Cassiman J.J., Van den Berghe H., Marynen P. (1993). Opposite effects of growth hormone and estrogens on the pregnancy zone protein serum levels in children and adolescents. Acta Endocrinol..

[23] Xuan C., Li H., Li L.L., Tian Q.W., Wang Q., Zhang B.B., Guo J.J., He G.W., Lun L.M. (2019). Screening and Identification of Pregnancy Zone Protein and Leucine-Rich Alpha-2-Glycoprotein as Potential Serum Biomarkers for Early-Onset Myocardial Infarction using Protein Profile Analysis. Proteom. Clin. Appl..

[24] Levin A., Stevens P.E., Bilous R.W., Coresh J., De Francisco A.L.M., De Jong P.E., Griffith K.E., Hemmelgarn B.R., Iseki K., Lamb E.J. (2013). Kidney Disease: Improving Global Outcomes (KDIGO) CKD Work Group. KDIGO 2012 clinical practice guideline for the evaluation and management of chronic kidney disease. Kidney Int. Suppl..

[25] Schwartz G.J., Muñoz A., Schneider M.F., Mak R.H., Kaskel F., Warady B.A., Furth S.L. (2009). New equations to estimate GFR in children with CKD. J. Am. Soc. Nephrol..

[26] Flynn J.T., Kaelber D.C., Baker-Smith C.M., Blowey D., Carroll A.E., Daniels S.R., Falkner B., Flinn S.K., Gidding S.S., Goodwin C. (2017). Clinical practice guideline for screening and management of high blood pressure in children and adolescents. Pediatrics.

[27] Wuhl E., Witte K., Soergelm M., Mehls O., Schaefer F., German Working Group on Pediatric Hypertension (2002). Distribution of 24-h ambulatory blood pressure in children: Normalized reference values and role of body dimensions. J. Hypertens..

[28] Daniels S.R., Kimball T.R., Morrison J.A., Khoury P., Meyer R.A. (1995). Indexing left ventricular mass to account for differences in body size in children and adolescents without cardiovascular disease. Am. J. Cardiol..

[29] Bode-Böger S.M., Scalera F., Ignarro L.J. (2007). The L-arginine paradox: Importance of the L-arginine/asymmetrical dimethylarginine ratio. Pharmacol. Ther..

[30] Flynn J.T., Urbina E.M., Brady T.M., Baker-Smith C., Daniels S.R., Hayman L.L., Mitsnefes M., Tran A., Zachariah J.P., Atherosclerosis, Hypertension, and Obesity in the Young Committee of the American Heart Association Council on Lifelong Congenital Heart Disease and Heart Health in the Young (2022). Ambulatory Blood Pressure Monitoring in Children and Adolescents: 2022 Update: A Scientific Statement from the American Heart Association. Hypertension.

[31] Fosheim I.K., Jacobsen D.P., Sugulle M., Alnaes-Katjavivi P., Fjeldstad H.E.S., Ueland T., Lekva T., Staff A.C. (2023). Serum amyloid A1 and pregnancy zone protein in pregnancy complications and correlation with markers of placental dysfunction. Am. J. Obstet. Gynecol. MFM.

[32] Wuhl E., Van Stralen K.J., Verrina E., Bjerre A., Wanner C., Heaf J.G., Zurriaga O., Hoitsma A., Niaudet P., Palsson R. (2013). Timing and outcome of renal replacement therapy in patients with congenital malformations of the kidney and urinary tract. Clin. J. Am. Soc. Nephrol..

[33] Tain Y.L., Lee C.T., Chan J.Y., Hsu C.N. (2016). Maternal melatonin or N-acetylcysteine therapy regulates hydrogen sulfide-generating pathway and renal transcriptome to prevent prenatal N(G)-Nitro-L-arginine-methyl ester (L-NAME)-induced fetal programming of hypertension in adult male offspring. Am. J. Obstet. Gynecol..

[34] Chebotareva N., Vinogradov A., McDonnell V., Zakharova N.V., Indeykina M.I., Moiseev S., Nikolaev E.N., Kononikhin A.S. (2021). Urinary Protein and Peptide Markers in Chronic Kidney Disease. Int. J. Mol. Sci..

[35] Cummins T.D., Korte E.A., Bhayana S., Merchant M.L., Barati M.T., Smoyer W.E., Klein J.B. (2022). Advances in proteomic profiling of pediatric kidney diseases. Pediatr. Nephrol..

[36] Liao W.T., Chen W.L., Tain Y.L., Hsu C.N. (2022). Complement Factor H and Related Proteins as Markers of Cardiovascular Risk in Pediatric Chronic Kidney Disease. Biomedicines.

